# NMR Characterization of Graphene Oxide-Doped Carbon Aerogel in a Liquid Environment

**DOI:** 10.3390/gels11020129

**Published:** 2025-02-11

**Authors:** Dávid Nyul, Mónika Kéri, Levente Novák, Hanna Szabó, Attila Csík, István Bányai

**Affiliations:** 1 Department of Physical Chemistry, University of Debrecen, 4032 Debrecen, Hungary; keri.monika@science.unideb.hu (M.K.); novak.levente@science.unideb.hu (L.N.); 2 Department of Inorganic and Analytical Chemistry, University of Debrecen, 4032 Debrecen, Hungary; 3 Institute for Nuclear Research, HUN-REN, 4026 Debrecen, Hungary; csik.attila@atomki.hu

**Keywords:** resorcinol–formaldehyde aerogels, carbon aerogels, pore size distribution NMR, relaxometry, cryoporometry

## Abstract

In this study, we report the findings of a morphological analysis of a resorcinol–formaldehyde (RF)-based carbon aerogel (CA) and its graphene oxide (GO)-doped version (CA-GO), prepared for possible applications as an electrode material. Beyond some electron microscopic and N_2_ sorption investigations, we mostly used NMR cryoporometry and relaxometry to characterize the gels in a wet state, as they are usually applied. The precursor RF polymer aerogel was prepared both with and without GO and was subsequently carbonized into carbon aerogel. Modifying the polymer aerogel using GO resulted in a larger variety of C-O bonds in both polymer aerogels. However, the most important changes occurred in the morphology of the carbon aerogels. NMR relaxometry revealed the highly hydrophilic nature of the pore wall of the RF polymer aerogels, as demonstrated by their uniform wetting behavior. The carbonization resulted in a mostly hydrophobic pore wall decorated by some oxygen-containing spots and a macroporous system. Doping with GO after pyrolysis resulted in spherical pores in the CA and cylindrical pores in the CA-GO, which is potentially a more promising material for electrochemical use than CA.

## 1. Introduction

Aerogels are at the center of current research due to their low density, high specific surface area, and increasing application potential. Carbon-based aerogels are applied as electrode and supercapacitor platforms, as well as adsorbents and catalysts for different purposes. Resorcinol–formaldehyde carbon aerogels (CAs) were first prepared, and are still prepared to a large extent, using resorcinol–formaldehyde polymer precursor aerogels (RFs) through pyrolysis [1]. The porosity, specific surface area, pore structure, and electrical conductivity of CAs, as well as their mechanical and chemical stability, can be easily tuned, making them promising candidates for a wide variety of applications [2,3,4,5,6,7,8,9]. The pore size distribution and hydrophilic/hydrophobic character of the surface—that is, the wetting properties—play important roles, since most applications take place in direct connection with a liquid (mainly aqueous) medium. The change in the size, the availability of the pores, and the adsorption capacity compared with the dry state are all vital information to know [10]. Highly hydrophilic precursor polymer RF gels become basically hydrophobic after pyrolysis; however, the carbon skeleton is still often decorated with heteroatom-containing functional groups. The heteroatoms are mostly oxygens in the form of C-OH, C=O, and C-O-C, which are primary binding/adsorbing sites for water molecules [11]. Thus, the oxygen content predominantly determines the wettability and hydrophilic character of the porous structure. High wettability is advantageous for adsorbents and catalysts because good contact between the phases is important, whereas lower wettability is advantageous for chemical and mechanical stability in a given liquid [2,10].

Classical methods—such as nitrogen adsorption and desorption measurements, mercury porometry, and microscopic techniques—are applicable for determining the size of micro- and mesopores, but only in their dry forms [12]. Non-conventional liquid-phase nuclear magnetic resonance methods, namely low-field NMR relaxometry and high-field cryoporometry, are applicable for this purpose [13,14,15,16,17,18,19,20,21,22]. Relaxometry can distinguish water in different states inside the porous material (e.g., adsorbed or confined in pores), while cryoporometry provides information about the pore shape and size in wet states [20,23,24,25,26,27,28]. Combining these methods can reveal the wetting mechanism of porous materials, from which information about the surface chemistry can be deduced. The theory and applications of these NMR methods are well established, and their applications for carbon aerogels have also appeared in the literature [29,30,31]. However, more experimental work is still important, both for the physical chemistry of CAs and to refine the theory of the NMR methodologies mentioned above.

Briefly, the NMR relaxation rate of protons in a liquid takes place through magnetic interactions with the environment. The efficiency of these interactions mostly depends on the tumbling motion of the given molecule and the magnetic properties of the environment. In pores, the liquid molecules that are close to the pore wall tumble slower and relax much faster than in the bulk phase. However, these molecules are exchanged quickly with the ones in the bulk, which tumble much faster if they are in contact with them. In the usual model, this exchange is so fast that a weighted averaged transverse relaxation rate (1/*T*_2_) is measured as follows:

(1) $$\frac{1}{T_{2}}=\frac{V_{\mathrm{sb}}}{V}\frac{1}{T_{2s}}+\frac{V_{b}}{V}\frac{1}{T_{2b}}$$

where *T*_2s_ and *T*_2b_ are the relaxation time constants of the liquid under the influence of the pore wall (exchanging with the bulk, with a volume represented by *V*_sb_) and the bulk, respectively (with a volume represented by *V*_b_). This model assumes that only these two types of water (*V* is their combined volume) are present in a pore when it is completely filled. Water molecules adsorbed on the pore wall but not in contact with the bulk are not detectable using NMR. V_s_. is the volume of liquid under the effect of the pore wall, measured independently of its contact with the bulk: *V*_s_. ≥ *V*_sb_ [16,31,32,33,34]. In relaxometry, water is the best test liquid because the usual applications of aerogels take place in water as sorbents or in aqueous solutions as catalysts [35].

NMR cryoporometry is a technical realization of the well-known thermoporometry method, based on the fact that liquid-state NMR can separately detect the amount of molten phase in the test material that is part of the solid–liquid equilibrium. It is based on the modified Gibbs–Thomson equation described in [20,36], as follows:

(2) $$T_{M/F}-T_{b}=\Delta T_{M/F}=K_{c}\frac{S}{V}=n_{M/F}K_{c}\frac{1}{r}$$

where *T*_M/F_ is the melting (M) or freezing (F) temperature of the test liquid embedded in the pore. *S* is the surface area, *V* is the volume of the pore, and *r* is a linear size equivalent to the radius for a sphere- or cylinder-shaped pore and the gap distance for a slit. *n_M/F_* is a small integer that can be correlated to the shape of the pore [20,37]. The *n_F_* (freezing) coefficient is 3, 2, and ½ for spherical-, cylindrical-, and slit-type pore geometries, respectively, while *n*_M_ (melting) is 2, 1, and 0, accordingly. *K*_c_ is the cryoporometry constant determined experimentally for the test liquids used. The melting and freezing point depressions are usually different; this phenomenon is called melting–freezing hysteresis. Their quotient, *n*_M_/*n*_F_ = Δ*T*_M_/Δ*T*_F_, varies between 0 (slit shape) and 0.67 (sphere), considering ideal geometries. This is calculated from experimental data and allows us to decide which of the *n*_M_ or *n*_F_ values can be used in Equation (2) [14,20,26,27,28].

The Gibbs–Thomson equation (Equation (2)) in this form is valid when the test liquid perfectly wets the surface of the porous material and the pores have the same shape and size; at minimum, the pore size distribution follows a narrow, normal distribution [38]. Over the last decade, strong requirements have arisen to characterize non-ideal systems or “disordered” porous materials using NMR cryoporometry given its relative experimental simplicity [21]. In parallel with new experimental evidence on disordered silicon and the continuous development of the theory of phase transitions in disordered systems, the origin of the hysteresis has been found to be more complex than described above [36,39,40,41,42,43]. Recently, an excellent review summarized the data and shed new light on the relationship between N_2_ sorption and thermoporometry techniques [36,40,44].

Graphene oxide (GO), discovered in 1859, is a group of thin, sheet-shaped molecules that are dispersible in alkaline aqueous media. GO has several types of oxygen-containing functional groups, allowing it to interact with monomers and prepolymers during the gelation processes [45,46]. According to earlier results, the morphology and surface chemistry of carbon aerogels (CAs) can be tuned by incorporating GO into RF polymer aerogels (RF-GOs) [46]. During the second phase of CA preparation—carbonization—the GO transforms into graphene. However, detailed characterizations have not been performed, as researchers have focused on the electrochemical and optical behavior of the product, reporting enhanced specific capacity and cycle stability for even 1 wt% GO [46,47]. GO has been applied to prepare graphene-doped carbon xerogels, resulting in mesopores interconnected with graphene lamella and leading to increased electric conductivity [47]. Meng et al. prepared graphene–carbon aerogels by using GO as a solid–base catalyst. The resulting aerogel was characterized in a dry state via nitrogen adsorption [48]. An increased GO ratio resulted in a larger BET surface and, consequently, higher conductivity. Guo et al. investigated the effect of GO on RF polymer aerogels and prepared RFs in which the GO was well dispersed and showed anti-shrinking activity [49]. Mesopores of 50 nm in diameter were found using N_2_ adsorption. They explained this effect with three factors: (i) the GO strongly interacts with the RF colloids, (ii) the GO decreases the pH of the precursor solutions, and (iii) the GO is a barrier between RF nanoparticles. Recently, Nagy et al. reported the effect of GO in higher ratios, up to 14 wt% [50]. They found that the morphology did not change after the addition of GO to the pre-polymerization medium, for either RF or CA. The mechanical properties were improved, and the shrinking level decreased during the supercritical drying and carbonization processes. However, the specific conductivity of CA increased linearly and doubled after applying up to 10% GO to the polymerization medium.

In this work, we prepared resorcinol–formaldehyde carbon aerogels with (CA-GO) and without (CA) adding GO to the polymerization medium. We aimed to determine how mesopore distributions change and how differently these aerogels behave in aqueous media. Both the polymer and carbonized aerogels were characterized via NMR relaxometry and cryoporometry, and we found different pore structures and relaxation behavior for the doped and non-doped carbon aerogels. Preliminary electrochemical measures show that the CA-GO could be a good cathode material for metal–air-based batteries.

## 2. Results and Discussion

### 2.1. Precursor RF and RF-GO Polymer Aerogels

#### 2.1.1. Electron Microscopic Data

The prepared graphene oxide (GO) was investigated via energy dispersive scanning electron microscopy (SEM-EDS) and XPS (X-ray photoelectron spectroscopy), making it possible to determine the elemental composition and chemical bond types of the materials. Figure S1 in the Supplementary Materials shows the formed GO layers. The SEM and XPS analyses confirmed that the sample is graphene oxide, as beyond the 73% carbon content, the oxygen content is relatively high, at 24%. The bond types are 51% C-C/C-H, 43% C-OH, and 5.5% C=O (Figure S2), similar to the compositions published in the literature [48].

**Figure 1:**
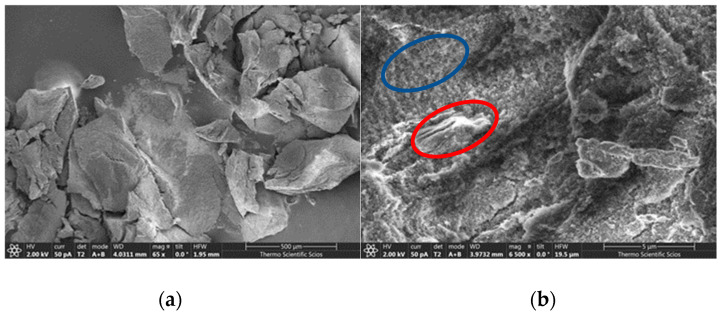
The SEM pictures of the GO doped RF aerogel, in 400 μm (**a**) and 1 μm resolutions (**b**). The GO layers are visible in the red ellipse while the intact RF aerogel structures are shown in the blue ellipse.

First, we partly characterized the precursor RF and RF-GO aerogels to inform further experiments and to confirm the presence of GO in their structures. Figure 1 is a SEM picture of the prepared RF-GO aerogel. Compared with SEM results for RF aerogels [51], GO layers in the aerogel were detected; therefore, a certain amount of GO was successfully embedded into the aerogel structure. Of course, the main part of the RF-GO structure is the same as that of the usual RF aerogels (Figure 1).

The SEM-EDS data show that the elementary composition of the RF-GO aerogel was 79%C and 21%O, and the XPS results show a high variety of bond types: the C-C/C-H ratio is 32%, the C-OH ratio is 32%, the C-O-C ratio is 29%, the COOH ratio is 5%, and the CO_3_^2−^ ratio is 3% (Figure S3). Although 2% GO did not change the carbon-to-oxygen ratio compared with the initial ratio, the wider variety of C-O bonding types and a lower ratio of C-C/C-H bonds indicate alterations caused by the GO in the RF-GO aerogel.

#### 2.1.2. Pore Size Distribution Results of RF Aerogels

The pore size distribution of the RF aerogel was determined via NMR cryoporometry using water as a test liquid to compare it with our RF aerogel, previously prepared according to the first reported method [1]. The only difference was the polymerization temperature, which was 75 °C in the present case instead of 85 °C. The melting–freezing curves of the water in the presence of the RF aerogel are shown in Figure S4 (Supplementary Materials). The Δ*T*_M_*/*Δ*T*_F_ estimation from the inflection point did not indicate any specific geometry, as a value of 0.56 was obtained. The calculated size distributions, assuming spherical and cylindrical ideal geometries, are shown in Figure S4. The real pore shape is probably a distorted sphere, closer to a cylinder, despite the regular sphere found for the RF aerogel in our previous paper [29]. The size of the precedent sample is also smaller at about 10 to 18 nm, considering that the geometry of the pore is between a perfect sphere and a cylinder. These observations support many reports in the literature concerning the high sensitivity of the morphology to preparation conditions [2,4,11,52,53]. We assume that applying the ideal geometric approximation for aerogels is possible because of their high porosity and Gaussian pore size distribution. This high porosity leads us to believe that the interconnecting channels or gates between the pores are very short and wide enough to avoid the ink bottle effect.

RF-GO aerogel was investigated in a dry state via N_2_ adsorption to check whether we obtained a porous aerogel. The adsorption isotherms and pore size distributions are shown in Figure S5: using the BJH method, the most probable pore size was 16 nm, as calculated from the desorption branch. Nitrogen adsorption experiments are suitable for examining micropores and small mesopores of up to approximately 30 nm [8,29]. Since we aimed to compare the aerogels through NMR data, we accepted the results of the sorption experiment as an indication of a porous structure, and we focused on the NMR results. NMR cryoporometry can provide information about larger mesopores and macropores of up to around a few hundred nm depending on the applied test liquid [20,21,54]. For example, using cyclohexane—for which the cryoporometric constant, *K*_c_, in Equation (2) is three times larger than that of water, with a melting temperature (280 K)—should lead to deeper insights into the mesopore structure. Illustrating the advantage of using cyclohexane, if the measurable smallest Δ*T* is 0.2 K (which is an underestimation), then even 900 nm pores can be determined in the case of an ideal spherical pore shape according to Equation (2). Therefore, we analyzed the pore structure of our aerogels with this technique using cyclohexane, following the suggestion of the very first NMR cryoporometry study [13]. We took advantage of its good wetting capability, owing to its low surface tension, which is likely necessary in the presence of GO in order to decrease the probability of the ink bottle effect. It is also important that the cyclohexane—being a paraffin—will not react with the carbon skeleton. We assume that it is possible to compare the two carbon aerogels based on ideal geometry approximations.

**Figure 2:**
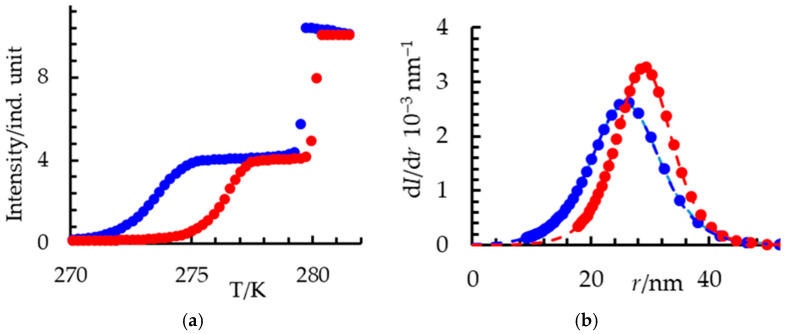
Cryoporometic (**a**) and size distributions curves (**b**) of RF-GO aerogel with test liquid as cyclohexane. • is the melting curve while • is the freezing curve.

Figure 2 shows the melting–freezing curves (a) and pore size distributions (b) of the RF-GO polymer aerogel. Using the inflection points of the curves, we could estimate the *n_M_/n_F_* ratio as 0.54; therefore, the shape of the pores is closer to an ideal cylindrical shape than a spherical one. Using the *K*_c_ value of cyclohexane (96 nm K), the most probable diameters are 56 nm and 66 nm, calculated from the melting curves and the freezing curve, respectively. This deviation from the pore size calculated based on N_2_ sorption may arise for two reasons. On the one hand, the cyclohexane may not be large enough to allow the molecule to reach micropores because they are not wide enough to be accessible; therefore, the observed pore size is different. On the other hand, the hydrophobic cyclohexane can reveal the mesopore structure of the RF aerogels, which is not “visible” in nitrogen sorption experiments, as it is above the upper detection limit [29,53]. A small freezing-point depression of 0.6 K, without a melting-point depression, indicates slit-like pores of about 70–80 nm. This is the distance between the individual aerogel particles immersed in cyclohexane.

#### 2.1.3. NMR Relaxometry of RF and RF-GO Polymer Aerogels

Using relaxometry on a porous material can provide information about the chemistry of the pore wall and the hydrophilic and hydrophobic character of the surface through the wetting mechanism. According to Fairhurts’ pioneering work, using standard reference material, the average pore size can be determined via relaxometry; however, we did not have an appropriate standard for our aerogels [31]. They were immersed in water as a test liquid because it is the liquid where most applications take place. Experimentally, the measurement was carried out by titrating the porous material with water, changing the volume fractions in Equation (1), and measuring the relaxation times. Equation (1) can be modified following Ardelean’s derivation developed for cylindrical pores [16,32,33]. When the wetting takes place uniformly, a complete adjacent surface liquid layer first forms in all pores (*V_s_* is its volume); then, the wetting continues layer by layer, covering this surface layer. In this way, the total surface layer is connected to the bulk; that is, *V*_s_. and *V*_sb_ are identical in Equation (1). In the other type of wetting, after the first layers form in all of the pores, the system takes up the water non-uniformly (“plug-like” for cylindrical pores); therefore, some surface layers are not covered by bulk water layers until complete filling is achieved. This means that V_s_. > *V*_sb_. In reality, the wetting occurs in a combined way; that is, *V*_sb_ = *V*_s_*f* ^(1−*k*)^, where *f* is the filling factor, which is 1 when all pores are completely filled. *F* is not calculated from the added water; it is determined using the NMR echo intensities [16,32,33]. As we deduced in Appendix A, following Ardelean’s concept, and given that 1/*T*_2*b*_ is negligible, the final equation is

(3) $$\frac{1}{T_{2}}=\frac{1}{T_{2b}}+\frac{\rho S}{f^{k}V_{0}}\;\;\;T_{2}\approx \frac{f^{k}V_{0}}{\rho S}$$

where *S* is the pore surface, *V*_0_ is the total volume of the pores, and *ρ* is the surface relaxivity defined by Equation (4)

(4) $$\rho =l\left[\frac{1}{T_{2s}}-\frac{1}{T_{2b}}\right]$$

where *l* is the thickness of the surface layer. The *k* in Equation (3) is an empirical constant characterizing the mechanism of the filling process. When *k* = 1, the water distribution is uniform, while at *k* = 0, it is “plug-like” for cylindrical pores [16,32,33]. For non-cylindrical pores, the “plug-like” model can be interpreted via Equation (5) in the Appendix A.

(5) $$x_{\mathrm{sb}}=\frac{V_{s}}{V_{0}f^{k}}$$

In this interpretation, when *k* = 1, the volume fraction of water under the effect of the pore wall (*x*_sb_) decreases, increasing the filling factor, *f*, characteristic for the uniform wetting. If *k* = 0 in Equation (5), the volume fraction of water molecules in the surface layer is constant during the whole wetting process. For cylindrical pores, this corresponds to cases when the volume of both the surface and bulk water increases but their ratio remains constant. However, for connected spherical pores, the wetting occurs pore by pore because, in each pore, the volume fraction of the surface water (*x*_sb_) is the same if the pore size is the same. Of course, a slight increase is possible if the porous system is not monodisperse, considering that the small pores are completely filled first [13,14,21]. In these experiments, disordered structures can also complicate the evaluation of the experimental data; therefore, calculating average pore sizes through relaxometry requires significant caution [13].

**Figure 3:**
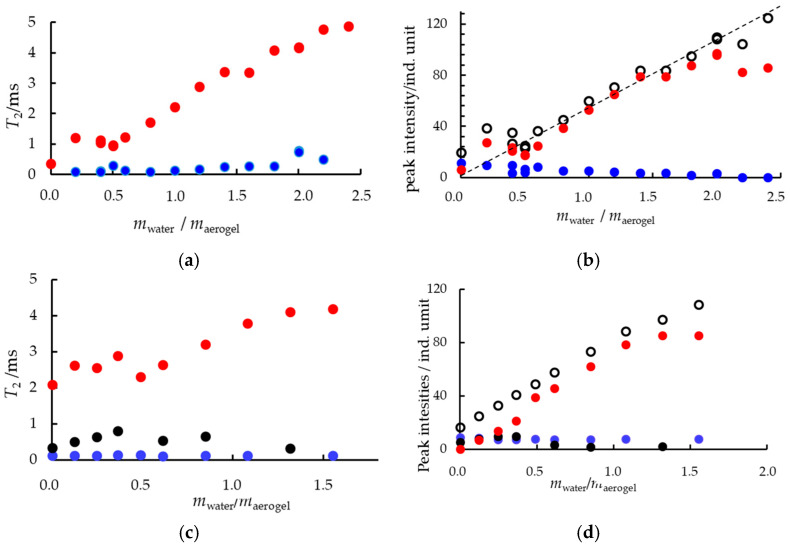
The measured relaxation times (**a**,**c**) and the peak intensities (**b**,**d**) as a function of the amount of water added to 1 g RF and RF-GO aerogel respectively. The red, blue and black dots correspond to the *T*_2_ values and the intensities of the characteristic relaxation domains. The empty black circles are the total intensities.

Our relaxation studies of the polymer aerogels (RF and RF-GO) demonstrate the effect of GO on surface chemistry and, to a certain extent, morphology. In each titration step, a relaxation distribution curve is obtained, showing the main relaxing water components. In Figures S6 and S7, the typical relaxation distribution curves of the RF and RF-GO polymer aerogels are shown during the course of titration with water. In Figure 3, the *T*_2_ values and corresponding intensity values during the titration of the polymer aerogels are shown. For the RF, two types of relaxation times are dominant (Figure 3a,b): fast ones, with *T*_2_ between 0.1 and 0.2 ms, and slower ones, with values between 1 and 5 ms. The intensity of the short relaxation domain is negligible after 0.2–0.3 g/g of added water. This indicates that “water puddles” form at and around certain hydrophilic surface groups either through water adsorption from the air or because of added water at the beginning of the wetting process [13,14,21]. The apparent *T*_2_ fluctuates within the range of 0.1–0.3 ms, and its amount decreases during titration (blue circles).

The ratio of these (probably hydrophilic) “puddles” becomes very low when the amount of water added increases until it becomes comparable to the amount of aerogel. The “puddles” cannot be adsorbed vapor because the adsorbed layer provides no NMR signal unless a second or further layers cover it, as the relaxation time of the adsorbed water is extremely short. A plausible explanation is that the water molecules adsorb to hydrophilic parts of the surface (probably the O-containing sites), with the following layers also preferring these points. The subsequent step depends on the position of these sites, which can be parts of micropores; these pores fill up completely, resulting in no (or little) change in the relaxation rate. The “puddles” on the walls of larger pores around the polar groups have *T*_2_ values of about 1 ms (red circles) at the beginning of titration. These “puddles” grow and merge on the surface of the pores and then thicken through the addition of more water, increasing the apparent *T*_2_. Figure 3 shows that the amount of this slower component continuously increases. After 0.3 g/g of added water, this component is dominant. Analyzing the plot of *T*_2_ over its filling factor is possible; according to Equation (3), the mechanism of the wetting process can be identified through the value of the *k* constant. The *f* values calculated from Figure 3 demonstrate up to 2 g/g of water content for the RF. After 2 g/g, the intensity is constant, indicating that the mesopores are completely filled.

**Figure 4:**
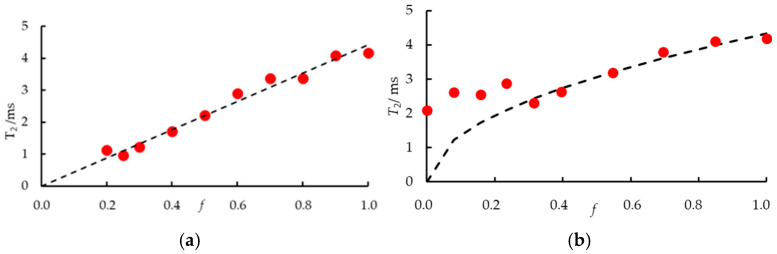
The apparent *T*_2_ of the RF (**a**) and the RF-GO (**b**) polymer aerogels as a function of the filling factor. *k* = 1 and the slope *m* = 4.42 ms for RF (**a**) while *k* = 0.5 and the slope is 4.38 ms for RF-GO (**b**).

As Figure 4a shows, applying Equation (3) to the RF results in a linear increase in *T*_2_ with an increasing filling factor. This means that the RF aerogel uniformly wets at the very beginning of the addition of water; a surface layer forms in the dominant pore from the *T*_2_ = 1 ms “puddles”. These puddles start to merge at 0.2–0.3 g/g of water. Subsequently, the water fills the pores uniformly, layer by layer. This uniform filling mechanism, considering the high surface tension of water, indicates the hydrophilic nature of the pore surface.

The wetting mechanism of the dominant pores in the RF-GO differs from that in the RF (Figure 3). At ~0.3 g/g of added water, three different “puddles” appear. Figure 3c shows that beyond the fast-relaxing “puddles” (blue), other fast-relaxing domains appear with *T*_2_ around 1 ms (black) and *T*_2_ 2–3 ms, indicating different types of hydrophilic spots on the pore surfaces. Presumably, the embedded GO layers (see the SEM pictures in Figure 2) create larger pores than those in the RF. In these pores, the hydrophilic spots are further away from each other, and therefore, the “puddles” (red) can grow larger than those in the RF, as their larger *T*_2_ (between 2 and 3 ms) indicates. After increasing the water from ~0.4 g/g, these “puddles” only partially merge and increase such that *V*_s_ > *V_sb_*. This is demonstrated in Figure 4b by applying the plot according to Equation (3). Since *k* = 0.5 results from Equation (3) for the RF-GO, the pore walls are not completely hydrophilic because of the larger distance between the puddles. There are parts where the water can wet the surface, but no further layers are formed; therefore, V_s_. is larger than *V*_sb._ The complete wetting starts when the “puddles” grow large enough and connect the spots. The slope (*m*) is a combination of the volume-to-surface ratio and the surface relaxivity. Considering that both aerogels are organic and the slopes are the same at 4.4 ms, we can state that either the surface chemistry or the size of the pores is not dramatically different. The distribution of GO in the structure moderately changes the size and the distribution of hydrophilic spots on the surface, as we assumed above.

### 2.2. Characterization of Carbon Aerogels

#### 2.2.1. Electron Microscopic Results

**Figure 5:**
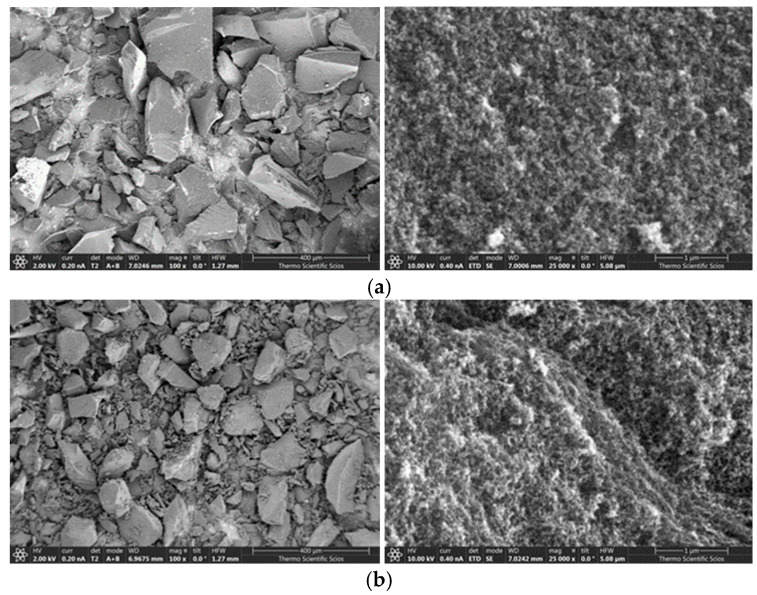
The SEM images of CA (**a**) and CA-GO carbon (**b**) aerogels. Magnification of 400 microns (**left**) and 1 microns (**right**) are shown. More SEM images are provided in Figures S11–S14 in the Supplementary Materials.

The two carbon aerogels are the main target of our investigations to determine the effect of GO on the morphology and behavior of CAs (CA and CA-GO) in water, i.e., the medium of potential applications. SEM pictures of both CAs are shown in Figure 5. At low magnification, the SEM pictures show smooth and homogenous surfaces, while at high magnification, the porous network is revealed. In both cases, the typical aerogel structure can be seen consisting of meso- and macropores on the nanoscale. The morphological difference between the two samples results from the embedded GO layers in the CA-GO. The analytical EM-EDS data show that the carbonization significantly reduces oxygen content to 3–4 mass% in both carbon aerogels compared with the polymer precursor gels, whereas the carbon content is 94–96 mass%. The similar composition of the CA and CA-GO is likely due to GO reducing into graphene, as a few earlier studies suggest [4,46,49,50,55]. In the Supplementary Materials, we show EM images for a few different enlargements from different locations.

#### 2.2.2. Pore Size Distribution Results of Carbon Aerogels

**Figure 6:**
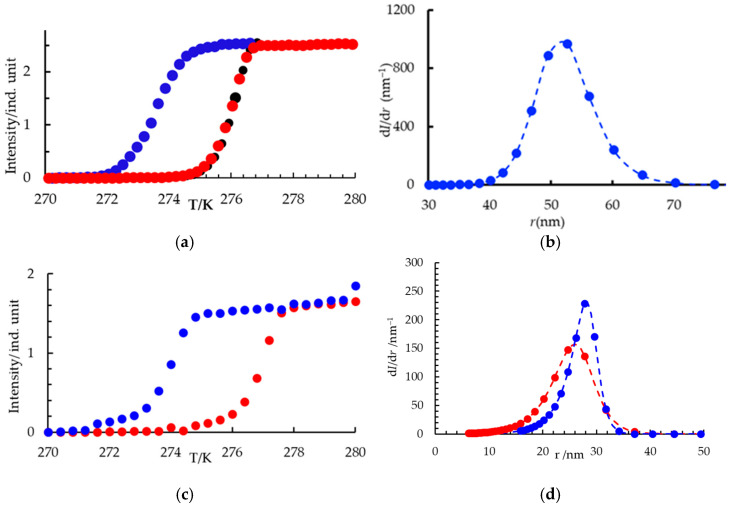
The melting-freezing curves of CA and CA-GO (**a**,**c**) using cyclohexane as test liquid in 4.98 g/g CA and 1.5 CA-GO. • the first melting curve (only for GO), • second (complete) melting curve while • freezing curve. The pore size distributions (**b**,**d**) calculated from the phase transition curves.

NMR cryoporometry experiments can reveal changes in porous structures. In Figure 6, the melting–freezing curves of the CA (a) and CA-GO (c) are shown. The *n*_M_/*n*_f_ values are 0.62 and 0.52, estimated from the inflection points of the CA and CA-GO, respectively. These values correspond to a rather spherical pore shape for the CA and a rather cylindrical shape for the CA-GO. The pore size distributions calculated from the melting–freezing curves are shown in the bottom diagrams of Figure 6.

The GO in the polymerization medium caused few differences in the pore shapes between the RF and RF-GO polymer aerogels. The original spherical pores described for RF [29] transformed into rather cylindrical pores after changing the polymerization temperature (see above). This observation highlights the sensitivity of aerogel formation to experimental conditions. Accordingly, the carbon aerogels made from the RF and RF-GO seem to preserve the pore shape of the RF and RF-GO during pyrolysis. The CA has spherical pores in good approximation, while the CA-GO has rather cylindrical ones. The pore sizes are also different. The CA has pores of about 100 nm, while the CA-GO has pores of about 50 nm (Figure 6).

**Scheme 1:**
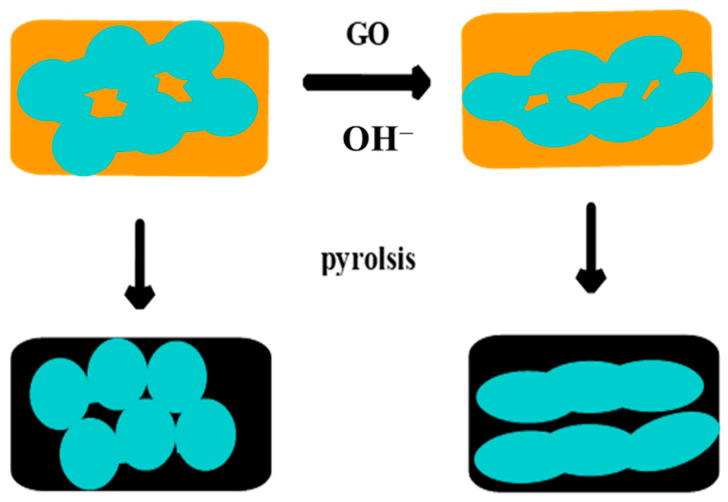
Graphical illustration of the formation of the suggested pore shape.

Since the applied amount of GO in the pre-polymerization medium is rather low, 2%, we originally assumed that GO acts in the polymerization and gelation phases by directing the reactions of the monomers or pre-polymers into another pathway. However, the effect of GO can hardly be deduced unequivocally from the cryoporometry results. The most critical parameter according to the literature is the resorcinol catalyst ratio, R/C [4]. Generally, Na_2_CO_3_ is suggested as the catalyst [4]. However, the catalyst is actually the OH^−^ ion, whose concentration is determined based on pH according to detailed studies [52,56,57,58]. Nevertheless, GO in the polymerization phase of synthesis possesses specific effects. It has been found to be a solid–base catalyst for the formation of RF aerogels [48] and an anti-shrinking agent and accelerator in the gelation process [49]. In general, GO has many effects on polymerization reactions [46]. The relevance of the spherical and cylindrical pore shapes deserves to be mentioned. A spherical pore shape can indicate spheres connected by short and thin channels (Scheme 1), whereas cylindrical pores indicate that the connecting channels have widened. This conforms with the cryoporometry calculation method and explains the non-perfect shapes that are often experimentally detected [20,27,28]. To further clarify the role of GO, a relaxometric analysis of the Cas was performed in aqueous media.

#### 2.2.3. NMR Relaxometry of CA and CA-GO 

**Figure 7:**
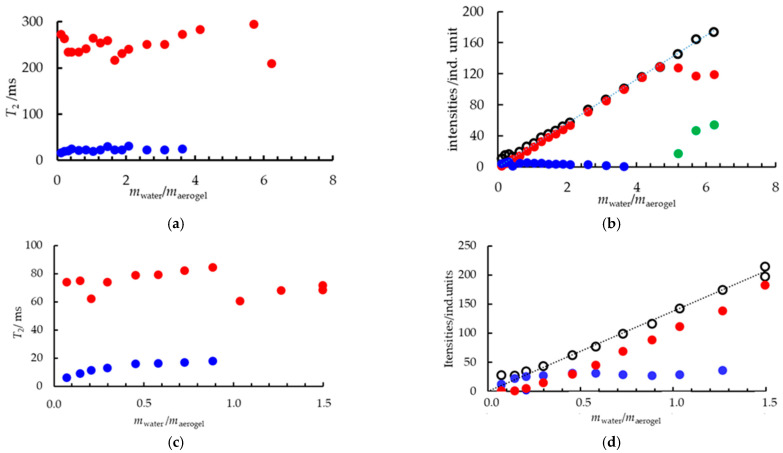
The measured relaxation times (**a**,**c**) and the peak intensities (**b**,**d**) as a function of added water to 1 g CA and CA-GO aerogel respectively. The red and blue dots correspond to the dominant *T*_2_ values (**a**,**c**) and the intensities of the characteristic relaxation domains (**b**,**d**). In green the possible bulk water intensities are indicated (**b**). The empty circles are the total intensities (**d**).

Figure S8 shows that two relaxation domains are dominant for the carbon aerogel (CA) prepared from the RF polymer aerogel. There is a time domain with a shorter relaxation time of around 20 ms and a longer one with relaxation times between 270 and 330 ms. A type of relaxing water with a relaxation time constant of more than 1 s appears only after adding 5 g of water to 1 g of CA, indicating that the pores are full. The apparent transverse relaxation times and intensity of the relaxation domains as a function of the amount of added water are plotted in Figure 7a,b.

Figure 7c,d show the results of the relaxometric titration of the CA-GO, and examples of typical relaxation distributions are shown in Figure S9. Both carbon aerogels show the same pattern, but with two striking differences. The first is the complete uptake of water in the mesopores at 4.6 g/g for the CA and at 1.3 g/g for the CA-GO. The second difference is the following: although both aerogels have two relaxation domains, the *T*_2_ of the longer one is three times larger for the CA (2–300 ms, red circles) than for the CA-GO (60–80 ms, red circles), while the shorter ones are very similar (blue circles).

For the CA, both relaxation time constants are independent of the added amount of water, indicating non-uniform wetting. Figure 7a also shows that the quantity of water belonging to the fast relaxation domain is negligible. The independence from the added water means that applying Equation (3) leads to *k* = 0. Via cryoporometry, we deduced that the mesopores (*d* = 100 nm) are spherical with a longer relaxation time; thus, the wetting mechanism should be a step-by-step filling of the pores. The short relaxation time domain is probably associated with micropores, but this is difficult to analyze due to the significant uncertainty of the values measured.

The pore size obtained for the CA-GO via cryoporometry is about 50 nm, and the melting–freezing hysteresis showed that the shape is very close to cylindrical. As noted above, the cylindrical shape can be interpreted as the narrow channels between the spheres becoming progressively wider and finally becoming long, continuous tunnels. This shape is very advantageous from an application perspective because the ions can diffuse more easily between cylindrical pores than between spherical pores [59,60,61]. Our pre-experiments on the use of these aerogels as electrodes seem to support this assumption. The *T*_2_ values of the CA-GO are much shorter than those of the CA, but the independence from the amount of water indicates that *k* = 0 here as well. This indicates a “plug-like” wetting mechanism. During the filling process, the volume-to-surface ratio is constant according to Equation (5) [16,32,33].

It is interesting to note that the slope, *m*, is zero for both carbon aerogels within the experimental error; in other words, *T*_2_ is independent of the filling factor, *f.* In this case, *T*_2_ is equal to *V*_0_/*Sρ* in Equation (3). Since *V*_0_/*S* is proportional to a linear size as *r*/*m*, where *m* depends on the shape of the pores and *ρ* is proportional to the surface relaxivity, we can say that *T*_2_ is determined by the size of the pore and the chemistry of the surface in this case. For carbon aerogels, the surface can only be different to a small extent, as both carbon aerogels are strongly hydrophobic. Therefore, the effect can be attributed to the difference in size. This agrees with the cryoporometry results and the amount of water completely filling the pores (Figure 7).

The intensity ratio of the fast-relaxing domain to the total intensity is larger in the CA-GO than in the CA; the plot according to Equation (3) shows a *k* of approximately 0.49, and *m* = 22.6 ms (Figure S10). Both types of wetting occur in this case, probably because of certain hydrophilic spots where the water can adsorb remain after carbonization. The slope is higher than that for polymer aerogels. According to Equation (3), this means a slower surface relaxation (*1/*T_2*s*_) or a larger size (*V*_0_/*S*)—or both—compared to the RF-GO. Carbonization results in a loss of oxygen, and therefore, the decreased surface relaxivity likely contributes to the effect to a larger extent than the change in pore size. Since the relaxation mechanism is probably dipolar, the rotation correlation time determines the value of *T*_2s._ The faster the rotation of the surface molecules, the longer the transverse relaxation time. The hydrophobic nature of the CA-GO’s surface results in the faster rotational motion of the water molecules compared with the hydrophilic surface of polymer aerogels.

These results support the widely accepted belief that the morphology of polymer aerogels is very sensitive to polymerization conditions. The most important factor is the resorcinol/catalyst (R/C) ratio according to the majority of the literature [2,4,11,52,62,63]. The application of GO as an additive has been investigated in the literature and has been found to be an anti-shrinkage additive for RF aerogels [49]. Our relaxometric results tend to confirm this finding: the RF-GO had larger pores than the RF polymer gel [34]. The effect of adding GO is complex because it contributes to polymerization as a catalyst and changes the morphology of the formed polymer gel [45]. Our cryoporometric and relaxometric experiments suggest that the usual spherical pores of the RF [29,34] change into cylinder-like ones through the addition of GO. For the carbon aerogels, the pore shapes are inherited, and pyrolysis strengthens these shapes. The CA has almost ideally spherical pores according to NMR cryoporometry, while the CA-GO has almost perfect cylinder-like pores, which is very advantageous for applications. However, one must be careful in attributing this to GO entirely, as the starting pH for RF polymerization was between 4 and 5 in contrast with RF-GO, where the pH was around 7 to prevent GO aggregation in the polymerization mixture. A lower starting pH (high R/C) results in larger pores in the RF aerogels according to the literature [4,11,62].

NMR cryoporometry and relaxometry provide valuable additional information for characterizing porous carbons [15,29,30,34]. In general, NMR data can predict behaviors that are very useful for later applications in liquid environments.

## 3. Conclusions

Carbon aerogels were prepared with (CA-GO) and without (CA) graphene oxide added during the polymerization phase. The main differences between these carbon aerogels were their pore size and pore shape. The CA had spherical pores of around 100 nm, while the CA-GOs were close to an ideal cylindrical pore shape, with a diameter of 50 nm. Importantly, we determined that it is very difficult to change only one parameter in aerogel preparation; therefore, discussions of the effects should be worded carefully. For example, to identify the role of graphene oxide, different characterization methods are required. NMR cryoporometry and relaxometry, two examples of these methods, provide information on morphology and surface characteristics in the presence of a liquid phase. However, comparisons with other methods are still needed through further experimental and theoretical efforts, mainly in cases of disordered systems. The advantage of using NMR techniques is that they are closer to the conditions where the usual applications take place. However, NMR methods are not a substitute for the classical methods; rather, they complete them, as suggested by the progress in this field [43,44].

## 4. Materials and Methods

### 4.1. Preparation of Graphene Oxide

The graphene oxide was prepared by oxidizing graphite (Alfa Aesar, Karlsruhe, Germany; size 7–10 μm) with KMnO_4_. In total, 0.5 g of graphite was suspended in 2.9 cm^3^ of concentrated sulfuric acid in an iodine flask. Under continuous stirring, an additional 20 cm^3^ of concentrated sulfuric acid (WWR, Debrecen, Hungary) was added to the sample. Following this, 2 g of P_2_O_5_ (Molar, Hungary) was dissolved in this liquid, and 1.5 g of KMnO_4_ was added in small portions to the intensively stirred mixture. The flask was always closed between the addition of the portions. In addition, we always waited for the temporarily formed manganese heptoxide (greenish oil-like liquid) to disappear. After 6 h of standing, we poured the mixture into icy water and removed the liquid phase with a centrifuge. The excess permanganate was reduced with H_2_O_2_ and washed away by changing the liquid phase until we reached a pH of about 2. The sample was then concentrated via diafiltration (with a 10 kDa filter at 3 bars of pressure) and washed two times with dilute sulfuric acid to remove the manganese ions and three times with distilled water until the pH rose to about 4–5. A concentrated suspension of *V* ≈ 100 cm^3^ was stored in a deep freezer (Lehel, Jászberény, Hungary) in 25 cm^3^ portions in plastic tubes. Before synthesis, the GO content was determined via lyophilization, measuring the mass of the remaining solid.

SEM-EDS images were created at the HUN-REN Nuclear Research Institute (Atommagkutató Intézet, ATOMKI, Debrecen, Hungary) by means of electronmicrospe of Bruker (Karlsruhe, Germany)

### 4.2. Preparation of the RF and CA 

Our concept was as follows: The first step in preparing the RF-GO hydrogel was adding the GO suspension to the resorcinol solution. The GO contains a remaining π electron system, making it hydrophobic, but the stacking interaction with resorcinol can form adducts in the pre-polymerization period, increasing the hydrophilicity of GO sheets. This process resulted in a stable suspension. We had to increase the pH up to about 7 to inhibit the aggregation of the GO sheets and ensure the gelation time was as short as possible. After these steps, we added formaldehyde and cured the sample at 70 °C.

The detailed method is as follows: In total, 2 g of solid resorcinol was dissolved in 12 cm^3^ of graphene oxide suspension (3.2 mg/g), and the pH was set to 7 by adding 0.1 mol dm^−3^ of NaOH solution to prevent GO sheet aggregation. The flask was closed, with only a narrow hole punched into the cap to prevent overpressure, and stored at 70 °C for two days, allowing gelation to occur. Subsequently, we changed the solvent to acetone, repeating the process twice a day for three days. Then, supercritical drying was executed using the usual carbon dioxide method [1].

The RF aerogel was prepared the same way but without adding GO to the mixture. The starting R/C ratio was set to 50 by adding Na_2_CO_3_ to the mixture. The rest of the processes were the same as described in the previous paragraph.

The RF aerogels were carbonized in a quartz tube reactor at 1073 K under an argon atmosphere [64]. The heat treatment lasted approximately 30 min in both cases. The formed carbon aerogels were crushed and dusted in order to conduct the cryoporometry and relaxometry experiments.

### 4.3. NMR Cryoporometry

Cryoporometric experiments were carried out on samples where the pores were completely filled with liquid. The aerogels were placed in 5 mm standard NMR tubes, and weighted amounts of cyclohexane (or, in one case, deionized Milli-Q water) were added and equilibrated for one day. A 360 MHz Bruker Avance I. (Bruker, Karlsruhe, Germany) NMR spectrometer was used with a 5 mm QNP direct probe head, cooled with dried air with a BCU05 cooling unit. The 1H spectra were recorded using a pre-optimized single-time spin-echo pulse sequence to reach the complet relaxation of the frozen liquid during the echo time (1 ms for water; 8 ms for cyclohexane) and thus eliminate its broad signal from the spectrum [20,27,28]. The experiments started with a fully frozen state, and the melting and freezing processes were observed between 265 and 285 K, with steps of 0.2–0.5 K. At each temperature, we waited 5 min for equilibration after the nominal temperature stabilized. Ethylene glycol and methanol were used to calibrate the temperature sensor prior to measurements, and we checked the melting point of the bulk solvent when it was observable [65]. MestReNova 9.0© was used for post-processing to determine the integrated intensity. Equation (2) was applied to calculate the pore radii from the melting and freezing point depressions. The *K*c values of water and cyclohexane were 30 nm K and 90 nm K, respectively. The shape of the pores was determined via melting–freezing hysteresis [20,27,28,37]. The integral–pore radius functions were fitted to asymmetric logistic curves (like the Richards and Gompertz growth functions) using OriginPro 8.6©, and their analytical derivatives were applied to determine the pore size distribution curves [66].

### 4.4. NMR Relaxometry

The carbon and polymer aerogel samples were powdered, measured into 10 mm glass NMR tubes, and kept at room temperature before the NMR measurements. Then, water was added until oversaturation in several steps (from 0.1 g to 1.5–5 g of water relative to 1 g of aerogel). Titration ceased when the dust-like structure of the mixtures became mud-like. Notably, the wetted solid aerogels were powder-like and, thus, easy to homogenize. At each titration step, *T*_2_ values were determined using a Bruker MiniSpec MQ20 relaxometer (Karlsruhe, Germany) at 298 K. The CPMG pulse sequence, provided with the relaxometer software, was applied to the samples [67,68,69]. We used a 90-degree pulse length of around 2 μs and a relaxation delay of 2–5 s, determined based on the *T*_1_ relaxation times. The echo time varied between 0.08 ms and 0.3 ms in successive CPMG experiments, and 500–1000 echoes were recorded to reach more than 90% signal intensity decay, providing a good basis for parameter estimation. The relaxation time distribution was evaluated with the free software MERA 2.03 (Multi-Exponential Relaxation Analysis), provided by Vanderbilt University [70].

## Data Availability

The data presented in this study are available upon request from the corresponding author due to the large amount of data.

## Supplementary Materials

The following supporting information can be downloaded at https://www.mdpi.com/article/10.3390/gels11020129/s1: Figure S1: SEM images of the prepared GO. The enlargements are 65× (A), 1500× (B) and 12,000× (C); Figure S2: The composition from SEM-EDS data (A) and XPS (B) spectra of the GO prepared; Figure S3: The composition from SEM-EDS (A) and XPS (B) spectra of the RF-GO prepared; Figure S4: Melting and freezing curves of water in RF aerogel and the resulting pore size distribution; Figure S5: The nitrogen adsorption experiments on RF-GO polymer aerogels; Figure S6: Typical relaxation time distributions of water in RF polymer aerogel. Left to right: 0, 0.4, 1.0 and 1.4 g water in 1 g of RF aerogel. The fit was made by MERA (Multiexponential Relaxation Analysis) software using inverse Laplace transformation; Figure S7: Typical relaxation time distributions of water in RF-GO polymer aerogel. Left to right: 0, 0.3, 0.9, and 1.3 g water in 1 g of RF-GO aerogel. The fit was made by MERA (Multiexponential Relaxation Analysis) software using inverse Laplace transformation; Figure S8: Typical relaxation time distributions of water in CA carbon aerogel. Left to right 0.1, 0.4, 2.1, and 5.2 g water in 1 g of CA aerogel. The fit was made by MERA (Multiexponential Relaxation Analysis) software using inverse Laplace transformation; Figure S9: Typical relaxation time distributions of water in CA-GO carbon aerogel. Left to right 0.1, 0.3, 0.5, and 1.5 g water in 1 g of CA-GO aerogel. The fit was made by MERA (Multiexponential Relaxation Analysis) software using inverse Laplace transformation; Figure S10: The apparent *T*2 of the faster relaxation domain CA-GO polymer gel as a function of the filling factor. *K* = 0.49 and *m =* 22.6 ms; Figure S11: EM images of CA-GO in different enlargements; Figure S12: EM images of CA-GO in 1 μm enlargement at different parts of the CA-GO aerogel; Figure S13: EM images of CA in different enlargements; Figure S14: EM images of CA in 1 μm enlargement at different parts of the CA aerogel.

## Appendix A

In our model, there are only two types of water molecules visible under NMR. One type is affected by the surface, with a volume fraction represented by *x*_sb_. The other type is bulk-like, with a volume fraction represented by *x*_b_. There is direct contact between the two types of water. The added water molecules which are on the surface but with no connection to the bulk are invisible under NMR. *T*_2s_ and *T*_2b_ are the relaxation time constants of the surface-affected water and the bulk, respectively. The measured relaxation time is

(A1) $$\begin{array}{l} \frac{1}{T_{2}}=x_{\mathrm{sb}}\frac{1}{T_{2s}}+x_{b}\frac{1}{T_{2b}}\\ x_{sb}=\frac{V_{\mathrm{sb}}}{V_{b}+V_{\mathrm{sb}}}x_{b}=\frac{V_{b}}{V_{b}+V_{\mathrm{sb}}} \end{array}$$

Since *x*_b_ + *x*_sb_ = 1,

(A2) $$\begin{array}{l} \frac{1}{T_{2}}=x_{\mathrm{sb}}\frac{1}{T_{2s}}+\left(1-x_{\mathrm{sb}}\right)\frac{1}{T_{2b}}=\frac{1}{T_{2b}}+x_{\mathrm{sb}}\left(\frac{1}{T_{2s}}-\frac{1}{T_{2b}}\right) \end{array}$$

We can define the filling degree, *f*, of the pore as the added volume of water divided by the pore volume:

(A3) $$f=\frac{V_{\mathrm{sb}}+V_{b}}{V_{0}}$$

where *V*_0_ is the pore volume. This value can be determined from the intensity of the NMR signal. The value of *f* is 0 when there is no water in the pores and 1 when the pores are completely filled. *V*_s_ is the total volume of the surface-affected water layer regardless of whether it is in contact with the bulk. The filling process is uniform when V_s_. = V_sb_ and non-uniform when

(A4) $$V_{\mathrm{sb}}=V_{s}f^{1-k}$$

where *k* is an empirically determined constant between 0 and 1. When *k* = 1, the whole surface layer takes part in the exchange process. When *k* = 0, the surface-affected water layer is proportional to the filling factor. With Equations (3) and (4), *x_sb_* can be rewritten as (5):

(A5) $$x_{\mathrm{sb}}=\frac{V_{\mathrm{sb}}}{fV_{0}}=\frac{V_{s}f^{1-k}}{fV_{0}}=\frac{V_{s}}{V_{0}f^{k}}$$

Through substitution into Equation (2), the relaxation is given as a function of the filling factor, *f*.

(A6) $$\frac{1}{T_{2}}=\frac{1}{T_{2b}}+\frac{V_{s}}{f^{k}V_{0}}\left(\frac{1}{T_{2s}}-\frac{1}{T_{2b}}\right)$$

We define the surface relaxivity for layer thickness *l* as

(A7) $$\rho =l\left[\frac{1}{T_{2s}}-\frac{1}{T_{2b}}\right]$$

The larger the thickness, the larger the relaxivity.

(A8) $$\begin{array}{l} \frac{1}{T_{2}}=\frac{1}{T_{2b}}+\frac{\rho V_{s}}{lf^{k}V_{0}}\\ \frac{V_{s}}{l}=S\\ \frac{1}{T_{2}}=\frac{1}{T_{2b}}+\frac{\rho S}{f^{k}V_{0}} \end{array}$$

In the last equation, if *k* is 0, then the relaxation time is constant with the addition of water. This means that according to Equation (5), the surface-affected volume, compared with the pore volume, is constant; that is, the pores are filling one by one. If *k* = 1, then the relaxation rate decreases with an increasing amount of water.
