# Supplementary material for: NMR Characterization of Graphene Oxide-Doped Carbon Aerogel in a Liquid Environment
Source: Gels. 2025 Feb 11;11(2):129. doi: 10.3390/gels11020129 (PMC11855333; doi:10.3390/gels11020129)
Supplement: Supplementary file 1 [file gels-11-00129-s001.zip › gels-3292889-supplementary.pdf]

Supplementary Material for

NMR Characterization of Graphene Oxide Doped Carbon Aerogel in Liquid Environment

Dávid Nyul[1*], Mónika Kéri[1] Levente Novák[1], Hanna Szabó[2], Csík Attila[3], István Bányai[1,*]

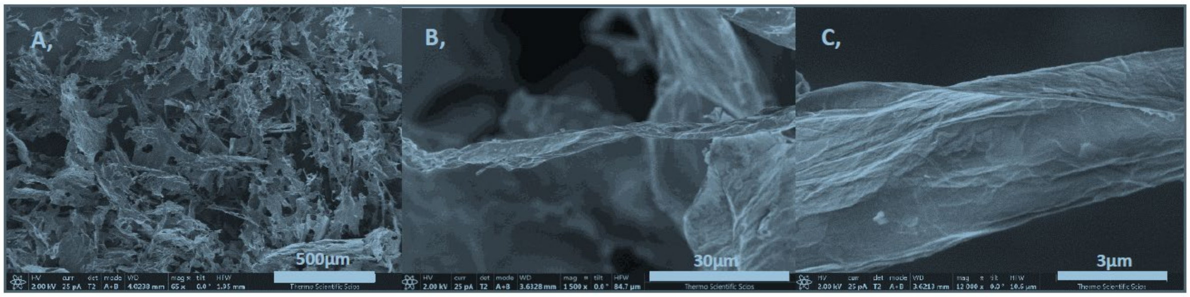

Figure S1. SEM images of the prepared GO. The enlargements are 65 x (A), 1500 x (B) and 12000 x (C).

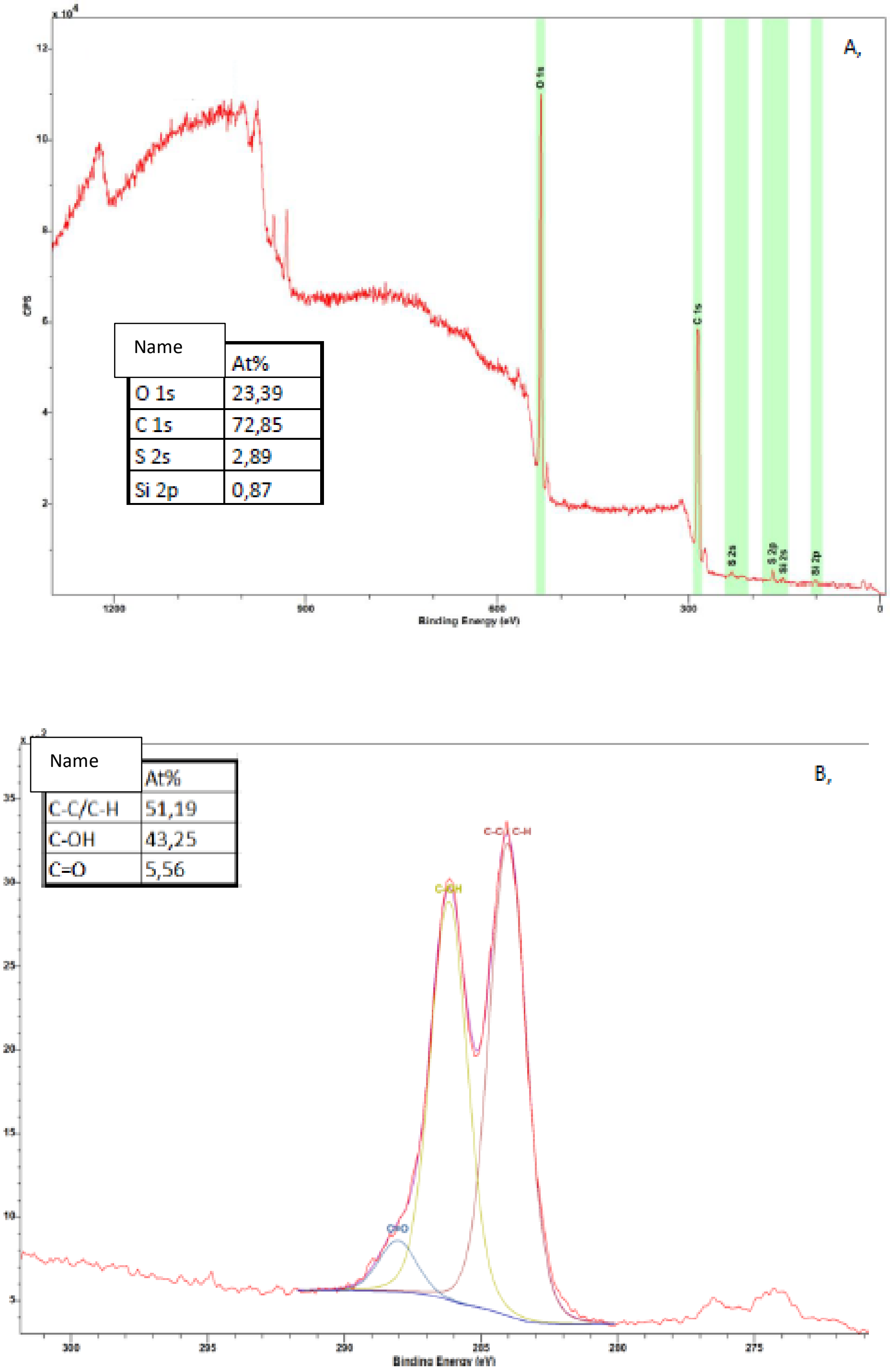

Figure S2. The composition from SEM-EDS data (A) and XPS (B) spectra of the GO prepared.

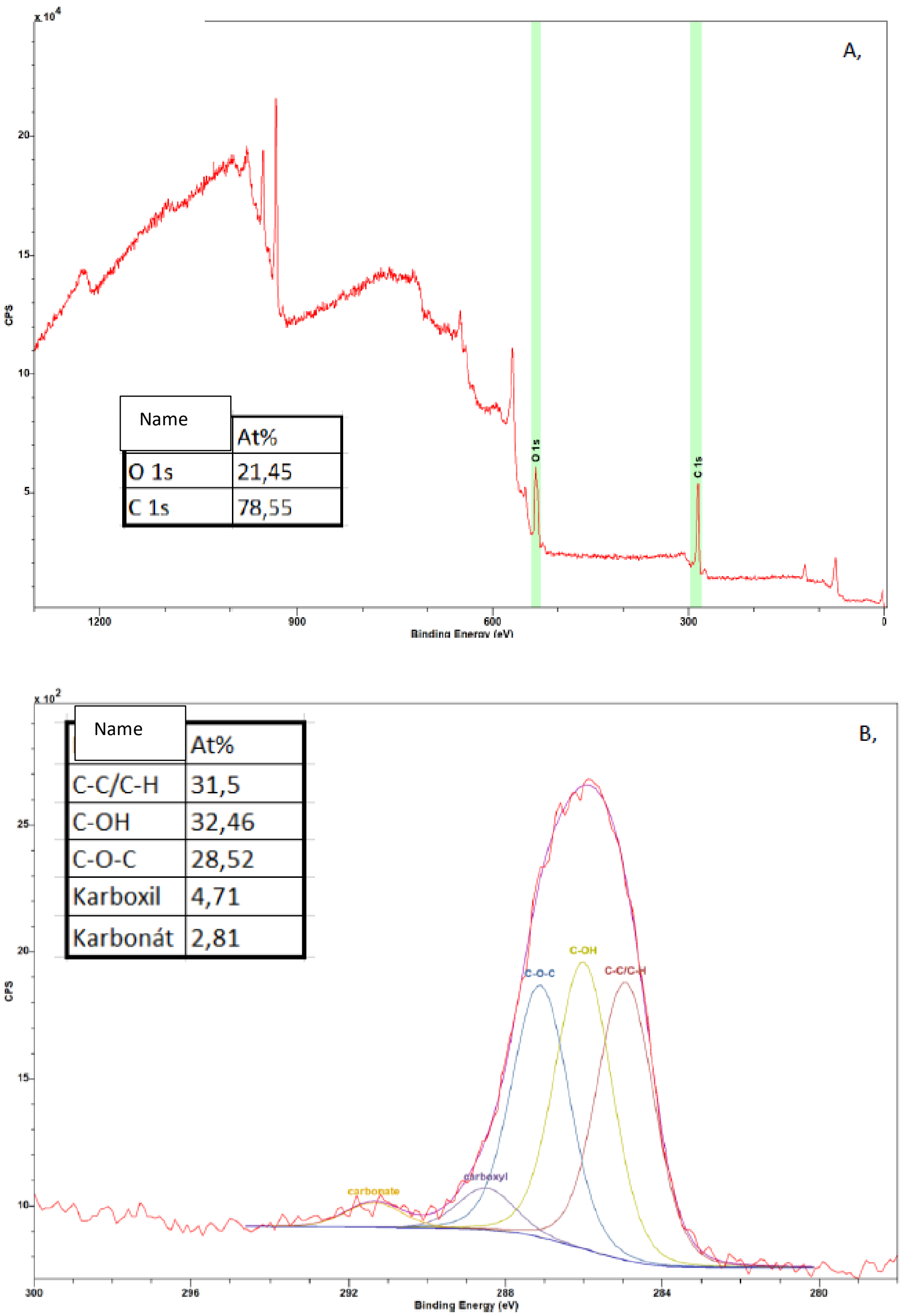

Figure S3. The composition from SEM-EDS (A) and XPS (B) spectra of the RF-GO prepared.

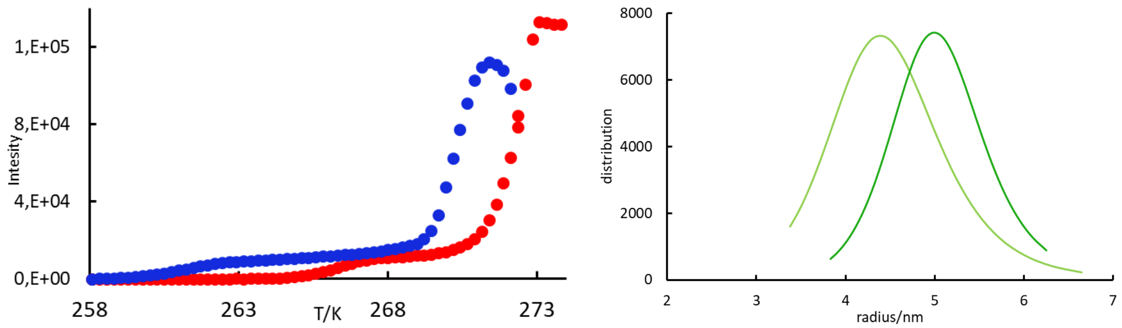

Figure S4. Melting and freezing curves of water in RF aerogel and the resulting pore size distribution.

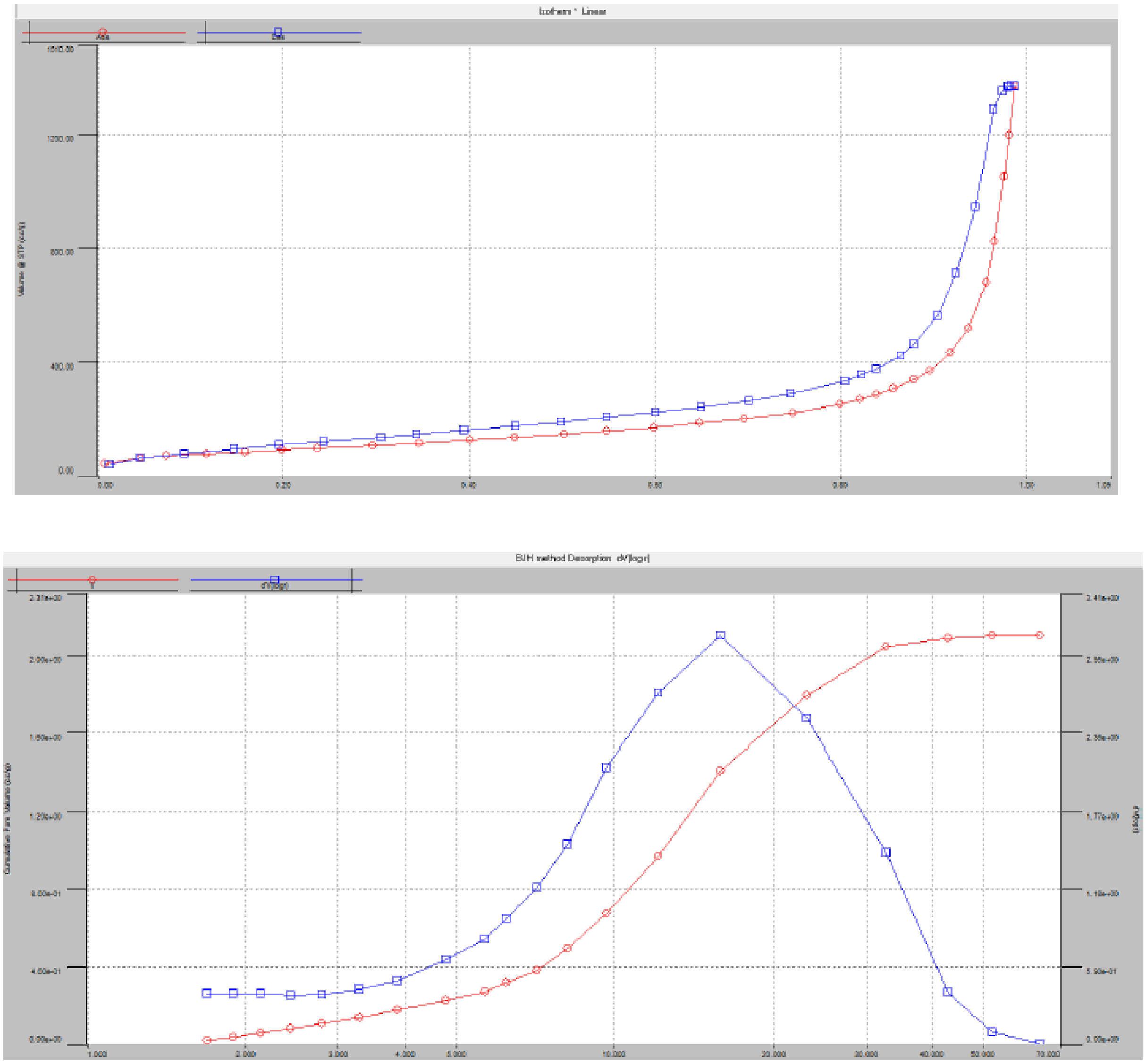

Figure S5. The nitrogen adsorption experiments on RF-GO polymer aerogels.

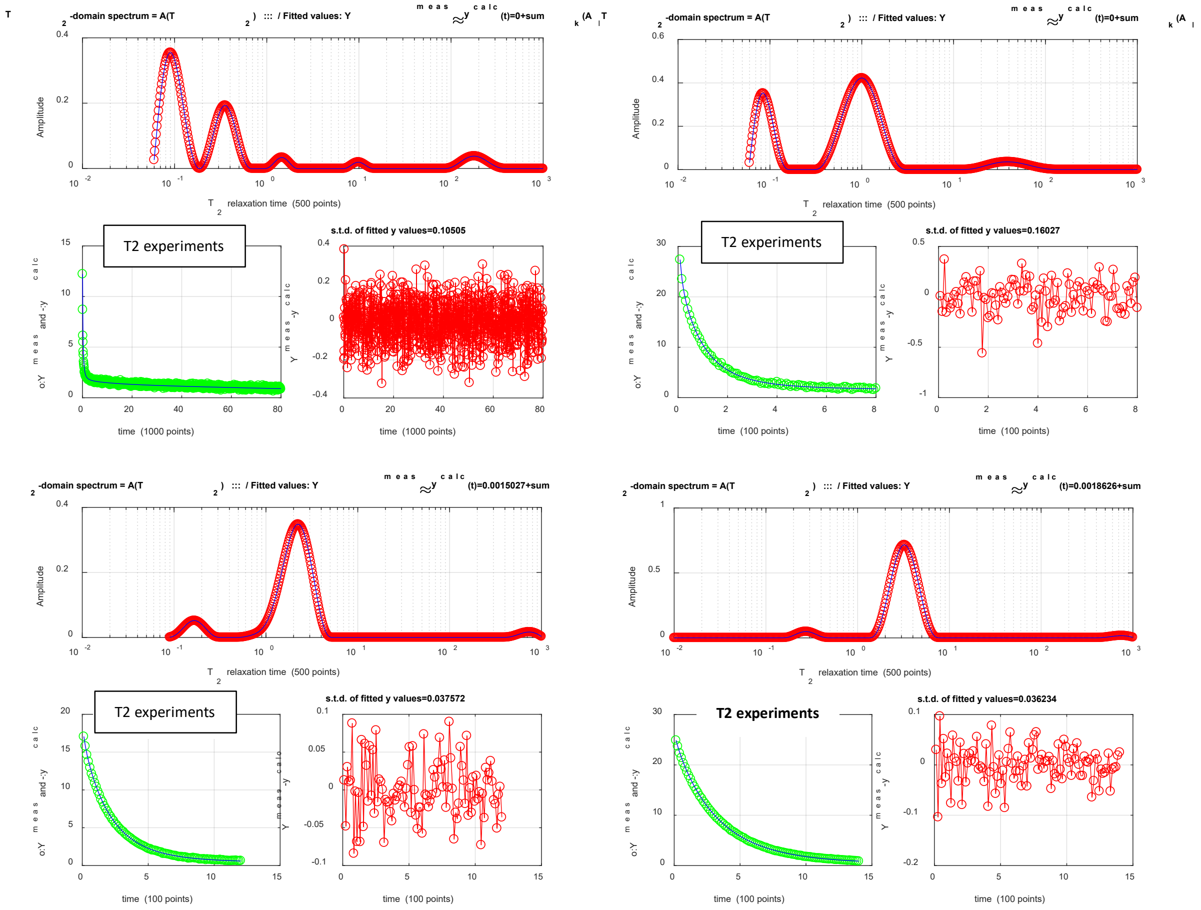

Figure S6. Typical relaxation time distributions of water in RF polymer aerogel. Left to right: 0, 0.4, 1.0 and 1.4 g water in 1 g of RF aerogel. The fit was made by MERA (Multiexponential Relaxation Analysis) software using inverse Laplace transformation.

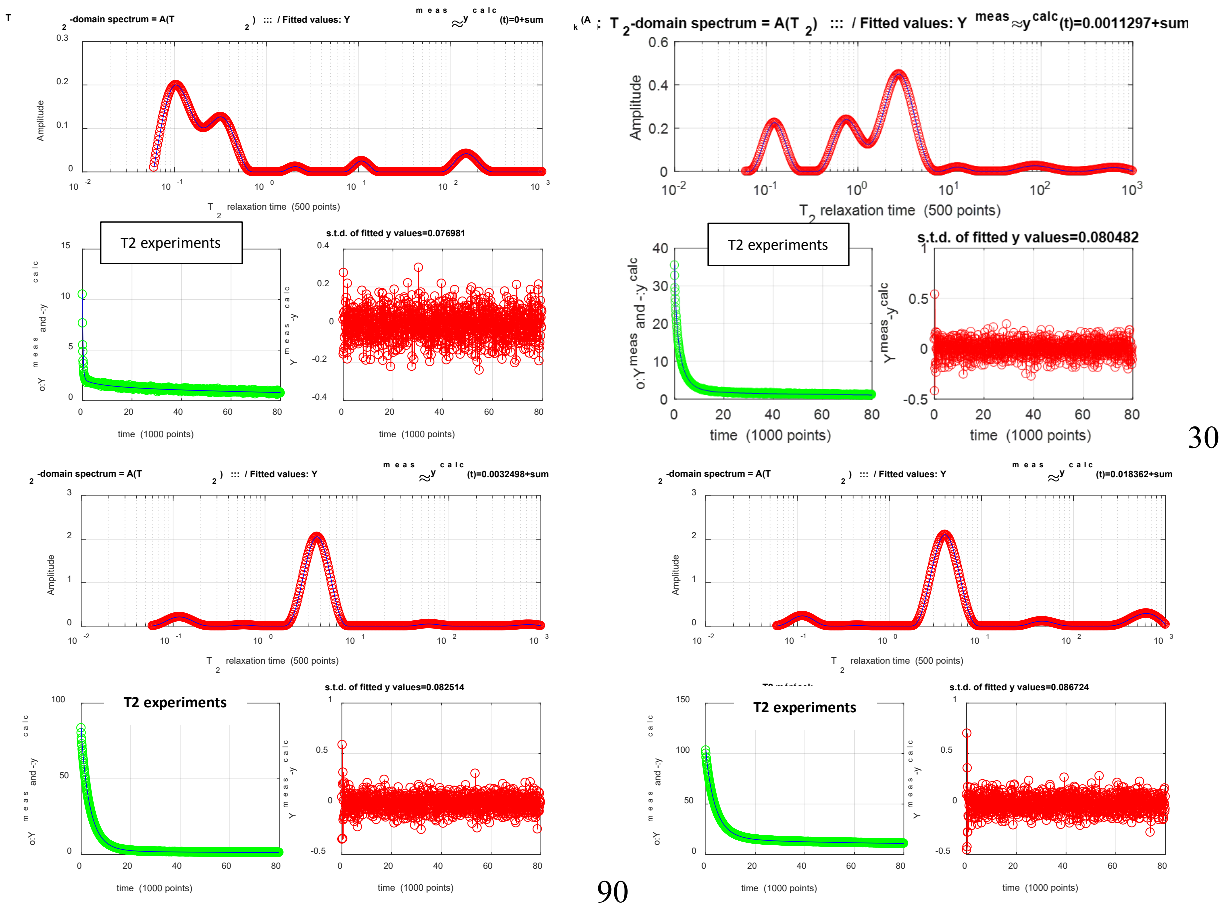

Figure S7. Typical relaxation time distributions of water in RF-GO polymer aerogel. Left to right: 0, 0.3, 0.9, and 1.3 g water in 1 g of RF-GO aerogel. The fit was made by MERA (Multiexponential Relaxation Analysis) software using inverse Laplace transformation.

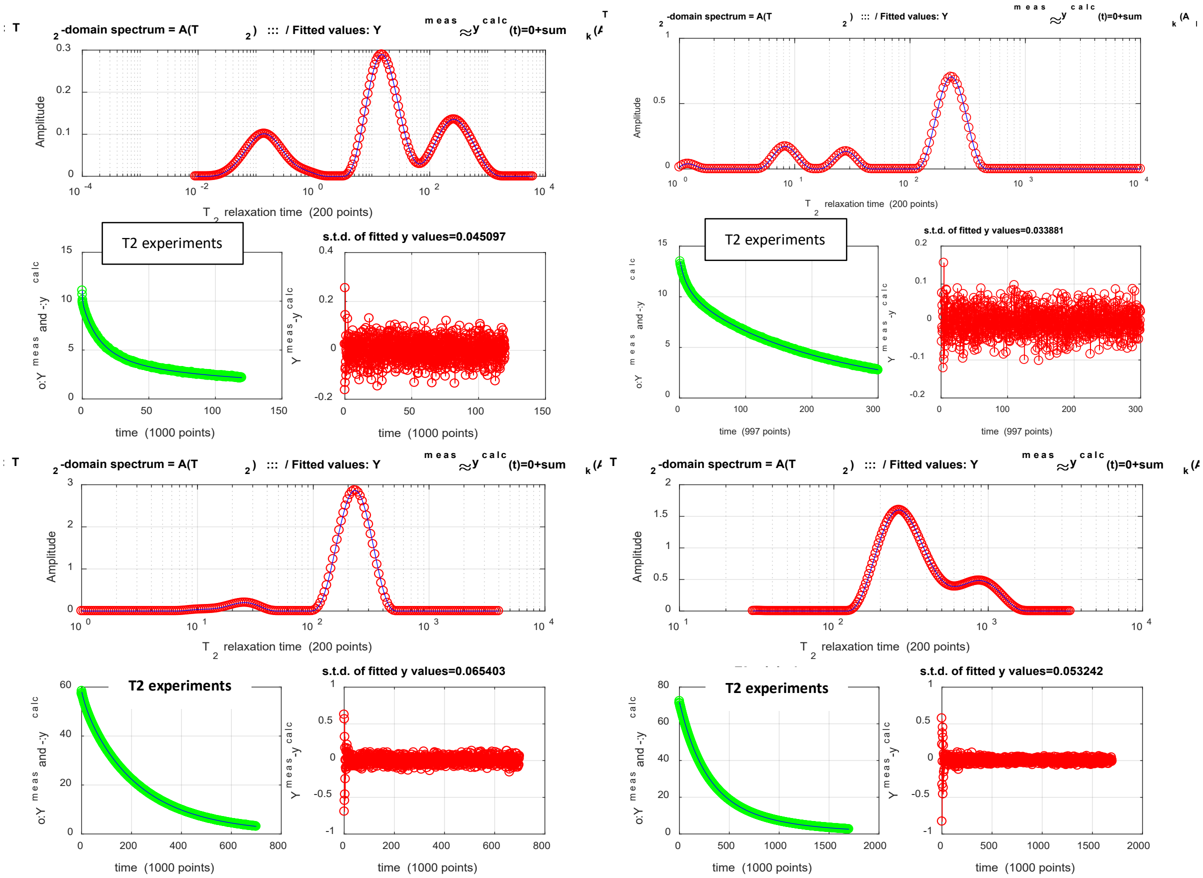

Figure S8. Typical relaxation time distributions of water in CA carbon aerogel. Left to right 0.1, 0.4, 2.1, and 5.2 g water in 1 g of CA aerogel. The fit was made by MERA (Multiexponential Relaxation Analysis) software using inverse Laplace transformation.

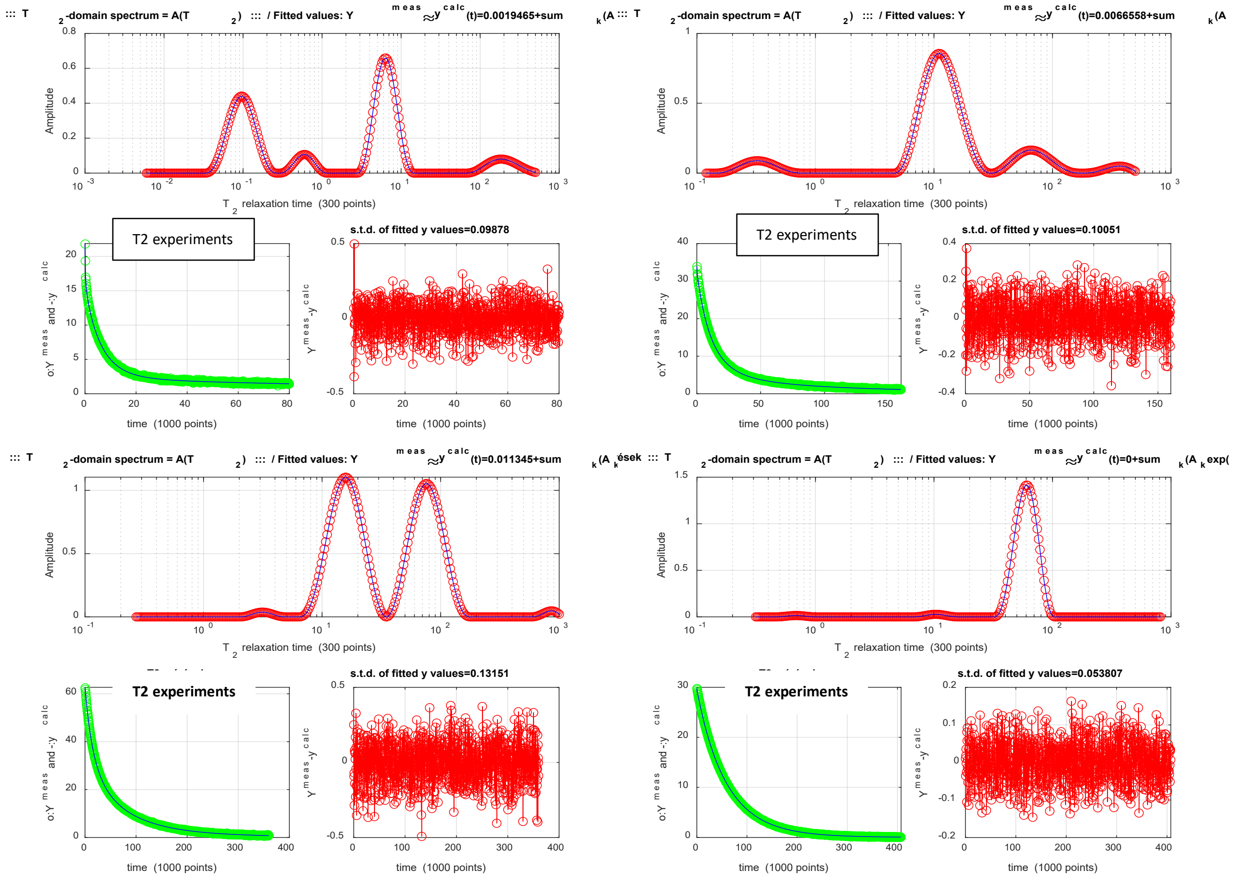

Figure S9. Typical relaxation time distributions of water in CA-GO carbon aerogel. Left to right 0.1, 0.3, 0.5, and 1.5 g water in 1 g of CA-GO aerogel. The fit was made by MERA (Multiexponential Relaxation Analysis) software using inverse Laplace transformation

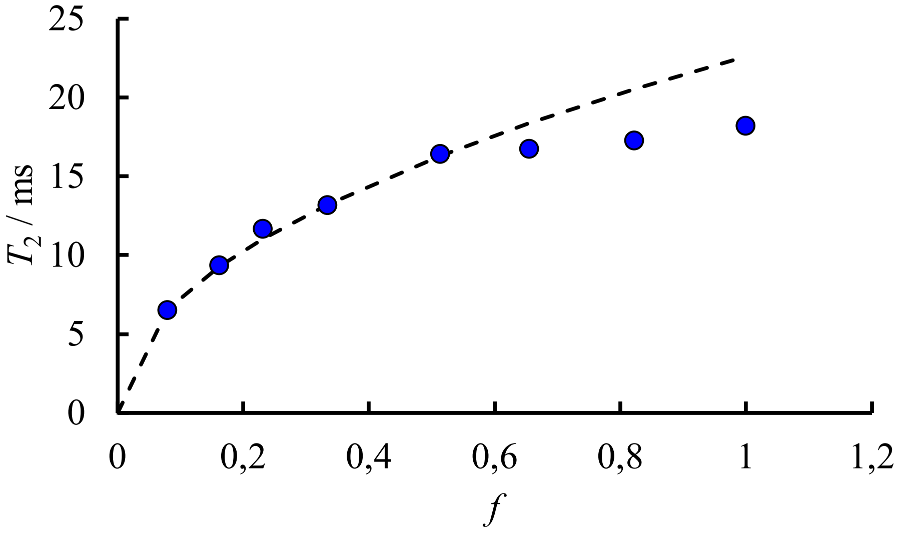

Figure S10. The apparent T_2 of the faster relaxation domain CA-GO polymer gel as a function of the filling factor. k=0.49 and m=22.6 ms.

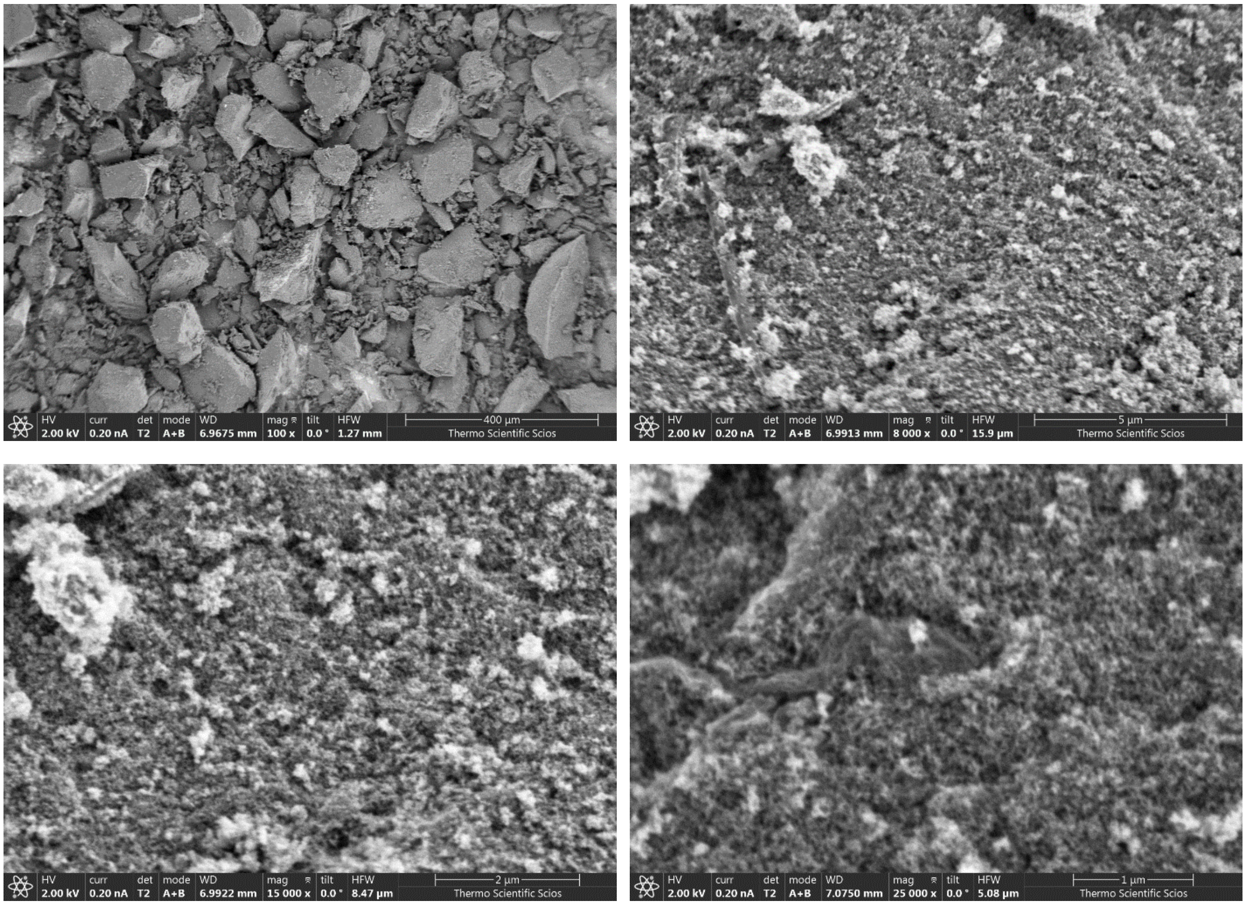

Figure S11. EM images of CA-GO in different enlargements.

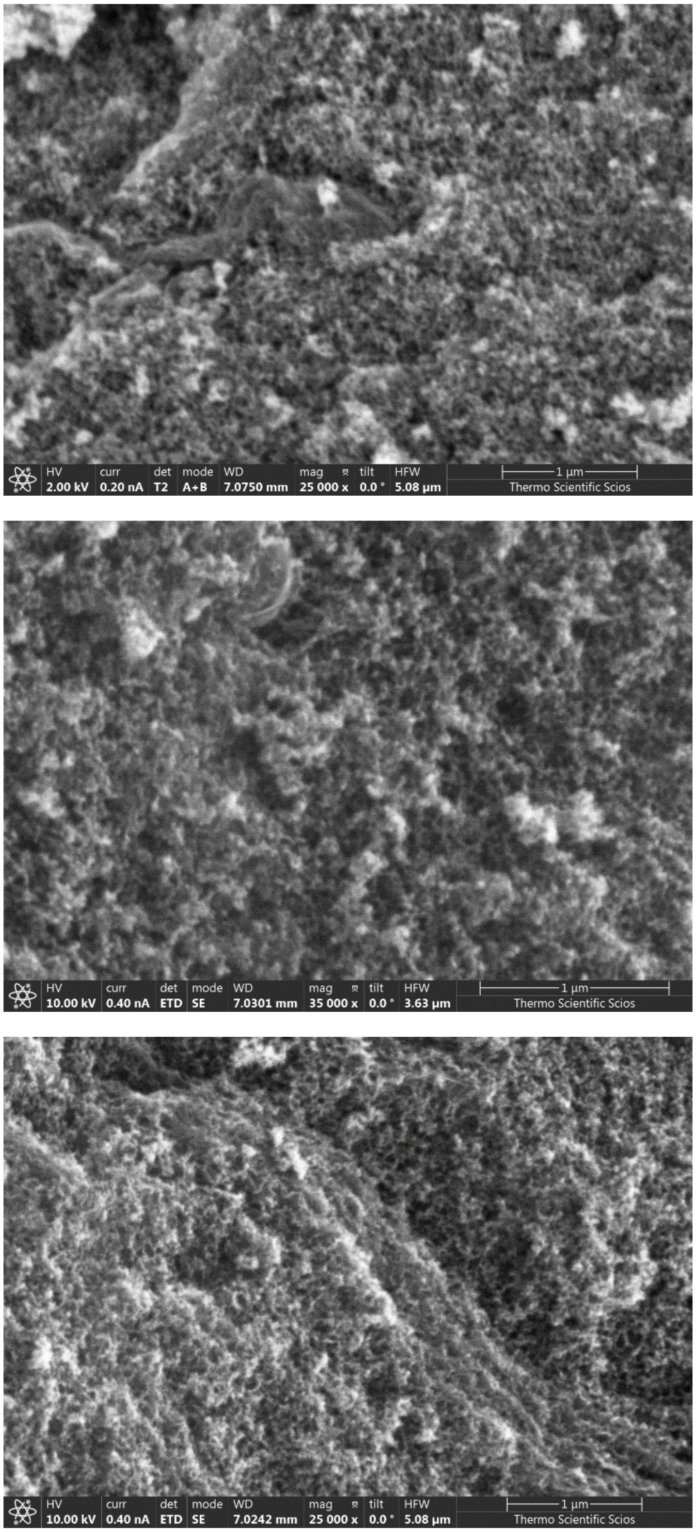

Figure S12. EM images of CA-GO in 1 μm enlargement at different parts of the CA-GO aerogel.

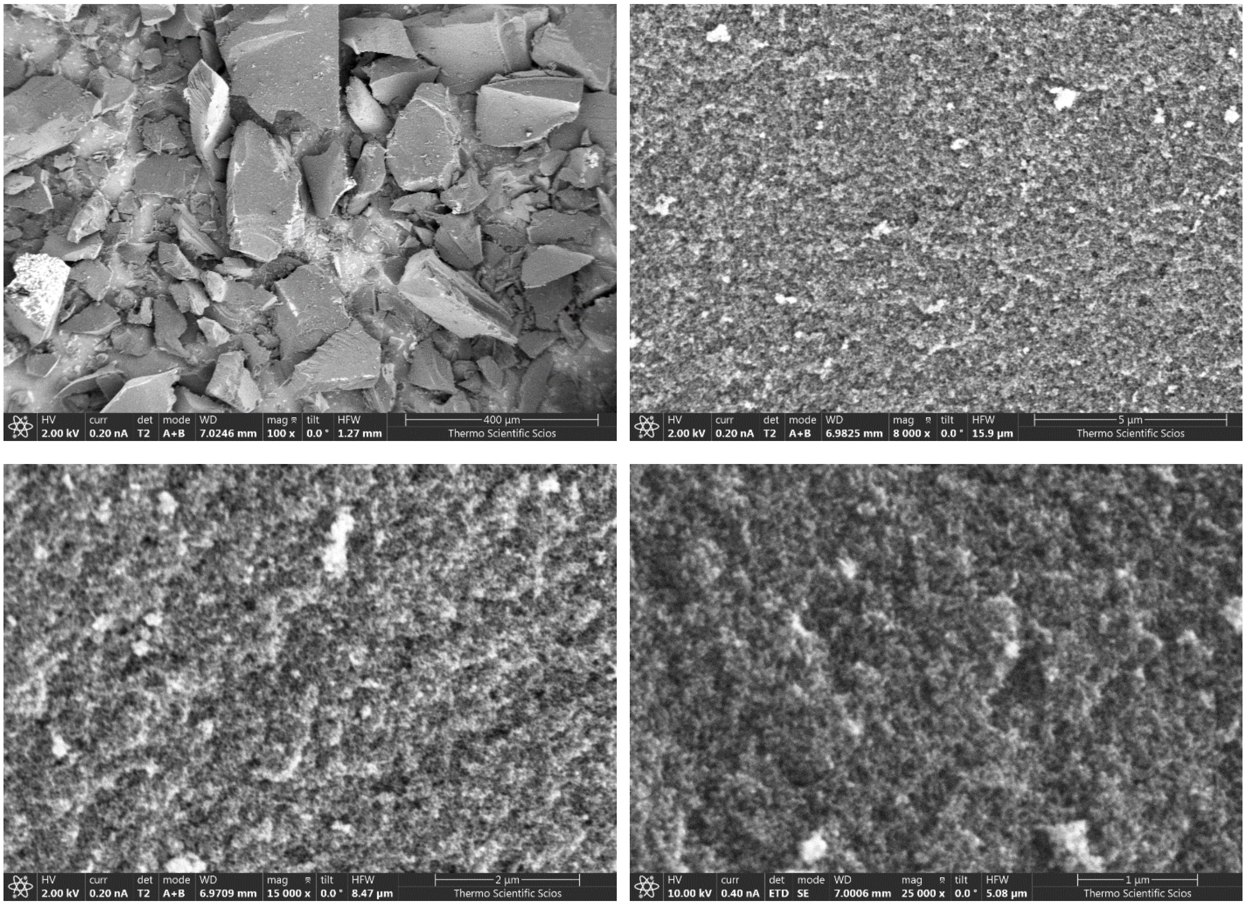

Figure S13. EM images of CA in different enlargements.

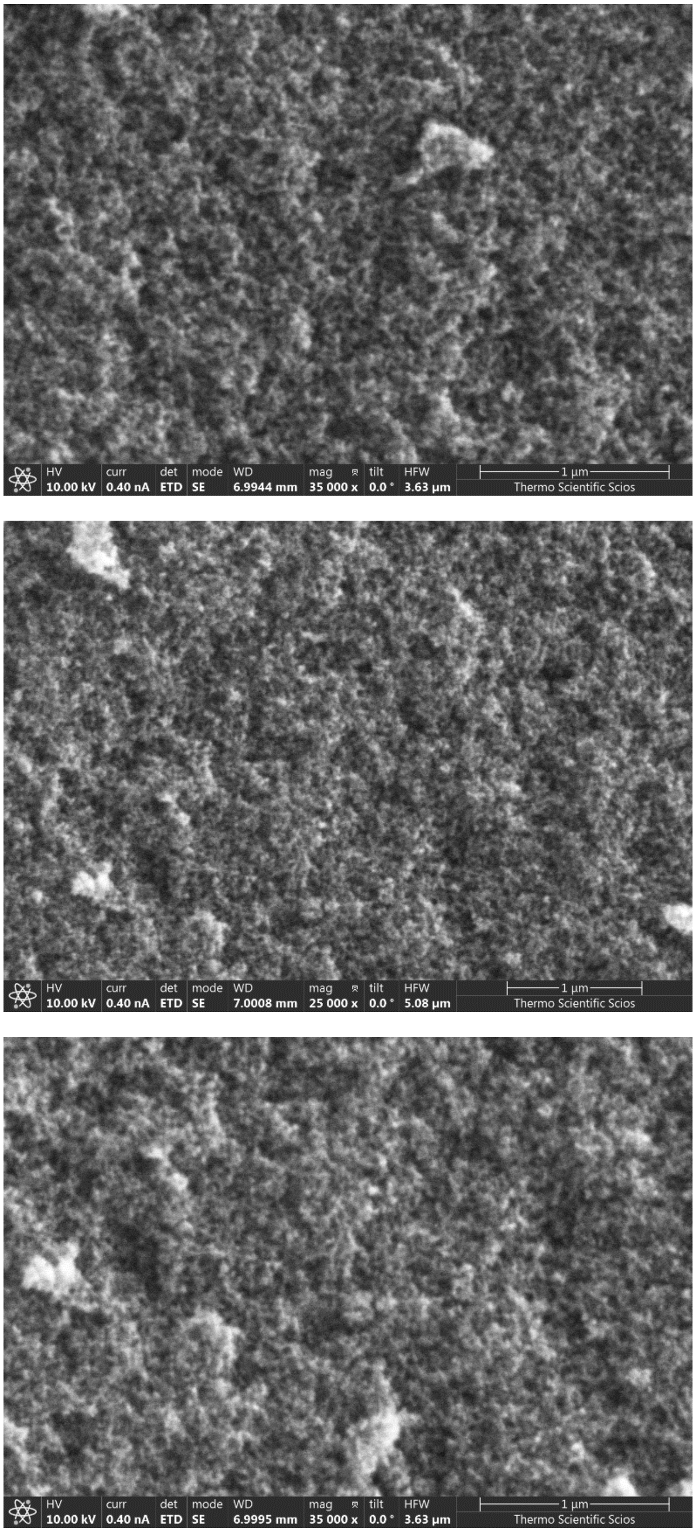

Figure S14. EM images of CA in 1 μm enlargement at different parts of the CA aerogel.
